# Protein-Targeted Degradation Agents Based on Natural Products

**DOI:** 10.3390/ph16010046

**Published:** 2022-12-28

**Authors:** Yan Li, Yi Jia, Xiaolin Wang, Hai Shang, Yu Tian

**Affiliations:** Institute of Medicinal Plant Development, Chinese Academy of Medical Sciences & Peking Union Medical College, Beijing 100193, China

**Keywords:** natural products, molecular-targeted drugs, targeted protein degradation agents, PROTAC, molecular glue

## Abstract

Natural products are an important source of drug lead compounds, and natural products with significant biological activity are constantly being discovered and used in clinical practice. At present, natural products play an important role in the targeted therapy of cancer, cardiovascular and cerebrovascular diseases, nervous system diseases, and autoimmune diseases. Meanwhile, in recent years, the rise of protein-targeted degradation technologies, such as proteolysis-targeting chimeras (PROTACs) and molecular glues, has provided a new solution for drug resistance caused by clinical molecular-targeting drugs. It is noteworthy that natural products and their derivatives, as important components of PROTACs and molecular glues, play an important role in the development of protein-targeting drugs. Hence, this review summarized the protein-targeted degradation agents based on natural products, such as PROTACs and molecular glues. More natural products with the potential to be used in the development of PROTACs and molecular glues as targeted protein degradation agents are still being investigated.

## 1. Introduction

As important sources of drug lead compounds, natural products play pivotal roles in drug discovery and development. It has been reported that, in the past 40 years, about 50% of the new drugs on the market came directly or indirectly from natural products. Natural medicines such as morphine, penicillin, aspirin, and paclitaxel are considered some of the most important active molecules affecting the course of human history. In recent years, drugs from natural sources have been found to have obvious advantages in antitumor, anti-infection, and neurological disease treatment. Moreover, the high probability of natural product discovery and structural diversity along with various properties provides more opportunities and possibilities for drug research and development [1,2]. To date, drugs obtained from natural products have been widely used in the clinical treatment of various diseases, such as paclitaxel for its anticancer ability; butylphthalide for its anti-cerebral ischemia ability; puerarin for its unique cardiovascular protection; the drug arsenic trioxide for the treatment of acute promyelocytic leukemia; and artemisinin, which saves millions of patients with malaria every year. Since the concept of tumor-targeted therapy was proposed, small-molecule-targeted drugs have been a research hotspot. With the continuous progress of isolation and identification technology, natural products with novel structures and significant activities have been discovered. Meanwhile, some new targets and mechanisms of the active compounds have been gradually clarified. Natural products and their derivatives, such as romidepsin and rapamycin, have become an important source of molecular-targeted drugs [3,4]. Compared with traditional chemotherapy drugs, molecular-targeted drugs have the advantages of low systemic side effects and good efficacy in disease treatment [5,6]. However, due to the cross-linking of signaling pathways resulting in drug resistance, the therapeutic effect of these molecular-targeted drugs has been limited to patients with the target gene aberration and is short-lived [7]. Because of this, in some cases, molecular-targeted drugs are not effective, and some proteins have poor targeting and druggability. Some proteins are resistant to molecular-targeted drugs, and others lack good targeting and druggability, such as disease-related transcription factors, skeleton proteins, and other proteins without enzymatic activity. In recent years, proteolysis-targeting chimeras (PROTACs) and molecular glue have evolved into two of the most important technologies for targeting protein degradation.

**Figure 1:**
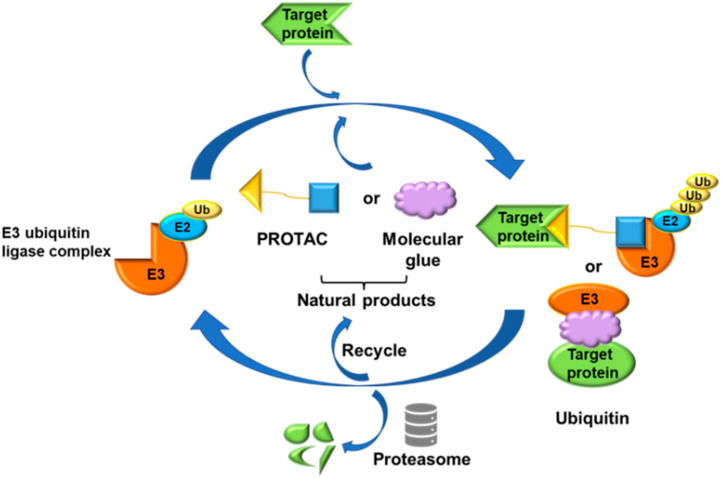
Mechanism of action of PROTAC and molecular glue technology.

The concept of PROTACs was first proposed by the team of Professor Craig M. Crews of Yale University and Professor Raymond J. Deshaies of California Institute of Technology. PROTAC technology is a targeted inducible protein degradation chimera formed by linking target protein ligands and E3 ubiquitin-ligase enzyme (E3) ligands through suitable linking chains. A PROTAC can simultaneously recruit the target protein and a specific E3 in vivo to achieve target protein ubiquitination degradation (Figure 1), which is a promising disease treatment strategy for target protein degradation via the ubiquitin-proteasome pathway [8,9]. Compared with traditional inhibitors, it has the advantages of high selectivity and potent activity and is expected to degrade non-generic targets and overcome drug resistance. Since the first polypeptide-based PROTAC molecule degrading MetAp-2 was reported in 2001, the technology has become an emerging strategy for target protein degradation using the ubiquitin-proteasome system. The earliest PROTAC was designed based on peptides, but its poor cell permeability and chemical stability meant that it did not have the potential to be developed into drugs. Researchers gradually shifted their focus to the development of small-molecule PROTACs, and they have been well-developed. To date, PROTACs have been successfully used in the degradation of different types of target proteins associated with a variety of diseases. In the PROTAC strategy, the following have been reported as targets: nuclear proteins, such as estrogen receptorα (ERα); cyclin-dependent kinases (CDKs); the breakpoint cluster region-c-abl (BCR-ABL) fusion protein; transmembrane proteins, such as human epidermal growth factor receptor-2 (HER2); c-mesenchymal-epithelial transition factor (c-Met); and cytoplasmic proteins, such as Brutons tyrosine kinase (BTK) and mouse double minute 2 (MDM2) [10].

Molecular glues are another class of small-molecule protein degraders acting via E3 ubiquitin ligases, the concept of which was proposed in the early 1990s. Although both molecular glue and PROTACs are protein degraders, they have different mechanisms of action and molecular structural characteristics. Molecular glue has high affinity and selectivity, so it can directly bind to proteins in order to form ternary complexes, and it can promote the dimerization or co-localization of two proteins [11]. By narrowing the distance between the target protein and the E3 ligase, molecular glue can induce the ubiquitination and degradation of the target protein, which is more in line with Lipinski’s five rules of drug-like (Figure 1). Molecular glue has a small molecular weight and good druggability, but it is difficult to design. According to recent research, some PROTACs designed target proteins play an anticancer role through the formation of molecular glue that can degrade new substrates. Some experts believe that the best PROTAC is made of molecular glue [12]. Therefore, in the design and verification of PROTACs, the two mechanisms should be considered comprehensively.

In recent years, natural products and their derivatives have played important roles in the design of PROTACs and molecular glues. The activities of natural products may occur due to interactions with multiple targets, but their affinities with one target are not very strong. The complexity of these mechanisms of action poses a huge challenge in identifying their targets. PROTACs and molecular glues can degrade target proteins effectively and specifically, and they bind to target proteins at lower dosages, which could be an efficient strategy for confirming the indetectable targets of natural products and for increasing their activities dramatically. Therefore, PROTACs and molecular glues that are designed based on natural products have great potential for protein degradation. This review summarized the progress of protein-targeting degradation agents based on natural products, such as PROTACs and molecular glues. The classes of these natural products include hormones, flavonoids, alkaloids, terpenoids, vitamins, microorganisms, and peptides. More natural products applicable for developing PROTACs and molecular glues are waiting to be explored. The toolbox of protein-targeted degradation agents will be significantly expanded.

## 2. Natural Products and Their Derivatives in PROTAC Design

### 2.1. Derived from Hormones

According to reports, not only is a malignant tumor one of the diseases with the highest morbidity in the world, but it also has a high mortality rate among major diseases, causing serious harm to human health. It has been difficult to achieve satisfactory results using traditional cancer treatment methods, such as surgery, chemotherapy, and radiotherapy, due to serious damage to the body and poor drug selectivity. At present, tumor-targeted therapy is the main direction of tumor therapy due to its specificity and targeting, and emerging protein degradation technologies, such as PROTACs, are also constantly opening up new fields of tumor-targeted therapy. Natural products contain endogenous compounds from plants and animals that play a positive role in regulating biological activities. At present, using natural products to fight cancer is the dominant direction of cancer drug development.

Breast cancer, as one of the most common malignant tumors, has seriously harmed the health of women all over the world. ERα is a member of the steroid hormone nuclear receptor family, and more than half of patients with breast cancer are ERα-positive [13]. Estrogen is a class of steroid compounds with a wide range of biological activities. Free estrogen enters target cells through passive diffusion or specific active transport, and it binds with the nuclear estrogen receptor (ER) to exert biological effects. Currently, antagonizing the interaction between estrogen and ERα is an important method for the treatment of breast cancer [14]. In addition, due to the existence of drug resistance, the use of selective estrogen receptor degraders (SERDs) to target the degradation of ERα is also an important direction in the development of breast cancer therapy. Estradiol (E2) is the most biologically active natural estrogen. Previous studies have shown that 17β-estradiol (Table 1 can be used as a ligand to recruit ER in the PROTAC design, inducing the specific degradation of ER through the ubiquitin-proteasome system [15]. Data from a previous study have shown that ligands linked to von Hippel-Lindau (VHL) E3 ligase via the C7α site of estradiol have higher affinity for estrogen receptors and more efficient ER degradation than C16α-based PROTACs, superior even to tamoxifen (Table 2). PROTACs, as an emerging strategy for protein degradation, have successfully achieved the ubiquitination of ERα [16]. Designing a new type of selective estrogen receptor degrader based on the PROTAC model is conducive to further expanding the PROTAC toolbox [17]. In addition, breast cancer growth and metastasis are dependent on angiogenesis, and ER is a potential anti-angiogenic target. PROTACs utilizing the E3 ubiquitin ligase pVHL ligand HIF-1α octapeptide combined with E2-Octa-[Ala] have endothelial-cell-targeting activity at low doses, and the use of ER-targeted PROTACs as probes for angiogenesis can be used to study the mechanisms of angiogenesis in animal models of diseases [18].

**Scheme 1:**
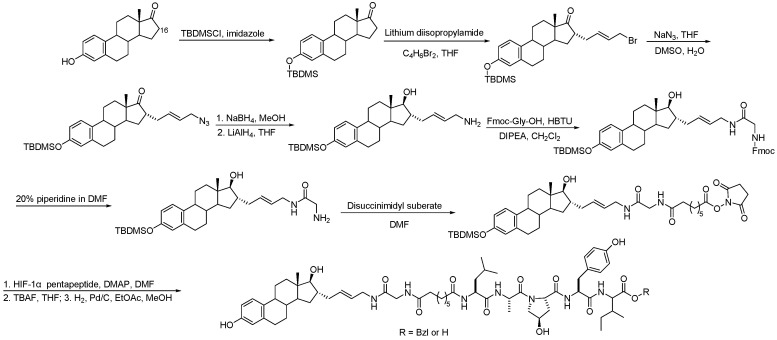
Synthesis of C16-derivatized estradiol–based PROTAC.

**Scheme 2:**
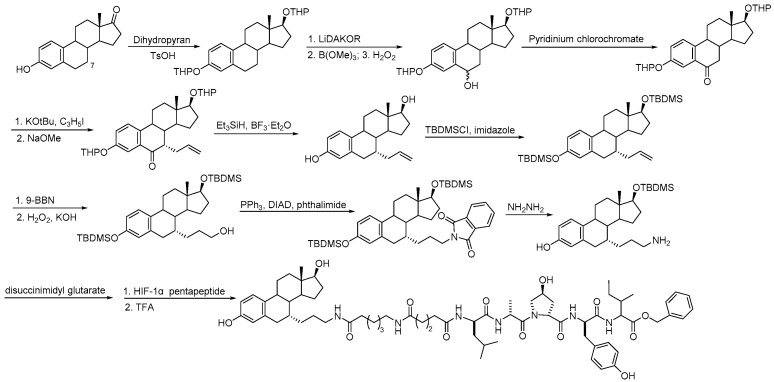
Synthesis of C7-derivatized estradiol–based PROTAC.

**Scheme 3:**
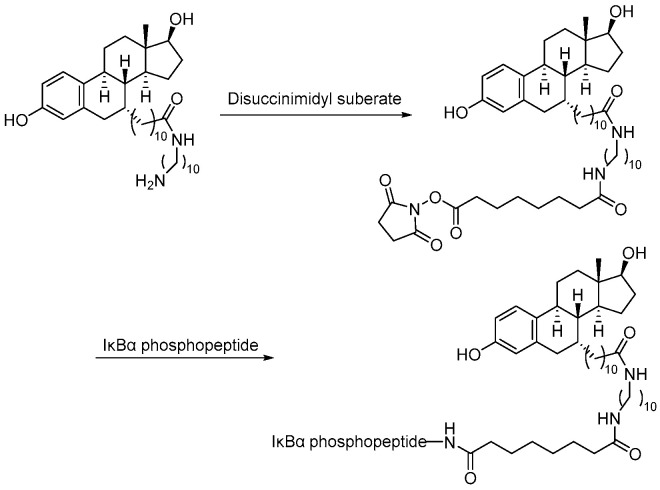
Synthesis of estradiol–based PROTAC.

**Scheme 4:**
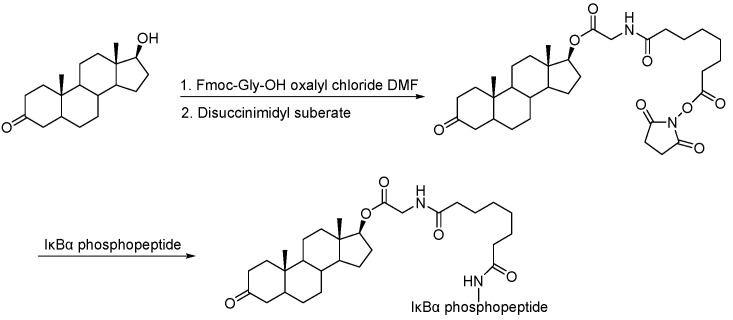
Synthesis of dihydroxytestosterone–based PROTAC.

Similarly, androgen receptor (AR) inhibitors are the main targeted drugs used for prostate cancer, and they are effective in metastatic castration-resistant prostate cancer (mCRPC). In the latest report in *Nature* on the sales of prostate cancer drugs, AR-targeted therapies account for 58% [19]. In recent years, a series of highly selective AR degradation PROTACs have been developed. Not only do these compounds effectively reduce AR protein levels in prostate cancer cell lines, but they also effectively inhibit cell growth in AR-positive breast cancer cell lines [20,21]. Dihydrotestosterone (Table 1) is a natural androgen that has made an important contribution to the pioneering work in the design and synthesis of PROTAC molecules for the degradation of AR. Preliminary experimental results have shown that AR degradation depends on the recruitment of E3 ligands and testosterone to bind to their respective targets, and the use of the green fluorescence protein (GFP) fusion protein technique provides a convenient method to monitor PROTAC-induced degradation (Table 2) [22,23]. The specific synthesis processes of the PROTACs discussed in this section are shown in Scheme 1, Scheme 2, Scheme 3 and Scheme 4.

**Table 1:** Natural products 1–2.

No.	Natural Products	Structure	Structure Classification	Source of Compounds	Reference
1	17β-estradiol	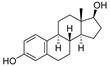	Sterides	Estrogen	[15,22]
2	Dihydroxytestosterone	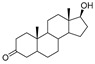	Sterides	Androgen	[22]

**Table 2:** Natural product targets 1–3.

No.	Target	E3	Cell Type	Reference
1	ERα	pVHL and SCF^β-TRCP^	MCF-7 cells	[15]
2	ERα	pVHL and SCF^β-TRCP^	293T cells	[22]
3	AR	SCF^β-TRCP^	293T cells	[22]

### 2.2. Derived from Flavonoids, Alkaloids, and Terpenoids

Apigenin (Table 3), a flavonoid natural product with anticancer activity, can directly interact with the ligand-activated transcription factor aryl hydrocarbon receptor (AhR) [24,25]. Using apigenin derivatives as ligands for recruiting AhR, the degradation of AhR was efficiently induced by the E3 ubiquitin ligase pVHL (Table 4) [26]. In addition, activated AhR also had E3 ubiquitin ligase activity [27]. Wogonin (Table 3) is a natural flavonoid extracted from the Chinese herbal medicine *Scutellaria baicalensis*, which has various biological activities, such as anti-inflammatory and antitumor activities, and it can improve diabetes [28,29]. Wogonin can directly bind to cyclin-dependent kinase 9 (CDK9) and selectively inhibit the activity of CDK9. A series of PROTACs targeting CDK9 can be designed based on wogonin. Previous results showed that the PROTACs obtained by linking wogonin and CRBN with triazole groups can selectively degrade CDK9 (Table 4) [30].

As the core skeleton of various natural products, oxindole spiro compounds (Table 3) have antimalarial, anticancer, antihypertensive, and other drug activities [31,32]. The E3 ubiquitin ligase MDM2 binds to p53 in order to ubiquitinate and degrade p53, and it is a major cellular endogenous inhibitor of p53. In human cancers, the overexpression of MDM2 leads to the inactivation of the tumor suppressor gene p53. Therefore, MDM2 can be used as a target of p53 activation, and the targeted degradation of MDM2-p53 can overcome the accumulation and potential adverse effects of MDM2 due to the activation of p53 [33,34]. The compounds MD-222 and MD-224, which utilize the previously reported oxindole spiro derivative MI-1061 as a ligand for recruiting MDM2 and a ligand for the ubiquitin ligase CRBN, are effective in mice and degrade MDM2 in vitro, and MD-224 efficiently induces the degradation of MDM2 in human leukemia cells at a concentration of 1 nM (Table 4) [35]. Researchers soon found that MG-277, obtained by removing the benzoic acid portion of the MDM2 inhibitor segment of MD-222, achieved significant cell growth inhibitory activity by inducing G1 to S phase transition 1 (GSPT1) protein degradation. This suggests that the designed phthalimide-coupled degradation agent can act as either a true PROTAC of the target protein or as a molecular glue (Table 4) [36]. Ursolic acid (UA) is a natural triterpenoid widely distributed in many plants (Table 3). Researchers connected UA and the CRBN ligand thalidomide through different linking chains and found that compound 1B with 3-Polyoxyether (POE-3) as the linking chain could inhibit the proliferation of A549, Huh7, HepG2, and other cancer cell lines and that it has significant in vitro antitumor activity (with an IC value of 0.23 to 0.39 μM) (Table 4) [37]. At the same time, the researchers confirmed that UA has the targeted binding properties of MDM2 and that the UA-PROTAC also promoted the expressions of P21 and PUMA proteins downstream of MDM2, thereby inhibiting the proliferation of A549 cells and promoting apoptosis.

Indirubin (Table 3), isolated from the traditional Chinese medicine Indigo Naturalis, is a new type of bisindole alkaloid antitumor drug that has a good curative effect on leukemia. By linking the natural product indirubin, a selective inhibitor of HDAC6, with the E3 ubiquitin ligase CRBN ligand pomalidomide, PROTAC compounds that can selectively degrade HDAC6 can be designed (Table 4) [38]. Targeting the STAT3 pathway has been shown to eliminate resistance to EGFR inhibitors in head and neck squamous cell carcinomas. Researchers designed and synthesized a PROTAC that specifically degrades STAT3 using the triterpenoid toosendanin compound (Table 3). This PROTAC has significant inhibitory activity in head and neck cancer and colorectal cancer tumor models (Table 4) [39]. Recently, based on the previously discovered molecular mechanism of broad-spectrum pentacyclic triterpenes inhibiting virus entry into host cells, Zhou and Xiao’s team linked oleanolic acid (OA) binding to influenza virus hemagglutinin protein (HA) to the CRBN and VHL ligands of E3 ligase by using PROTAC targeted protein degradation technology (Table 3) [40]. In this study, the PROTAC molecule was constructed to degrade hemagglutinin at both the molecular and cellular levels, and compound V3 effectively degraded the A/WSN/33 (H1N1) virus protein (DC_50_ = 1.44 μM) during viral replication (Table 4).

**Scheme 5:**
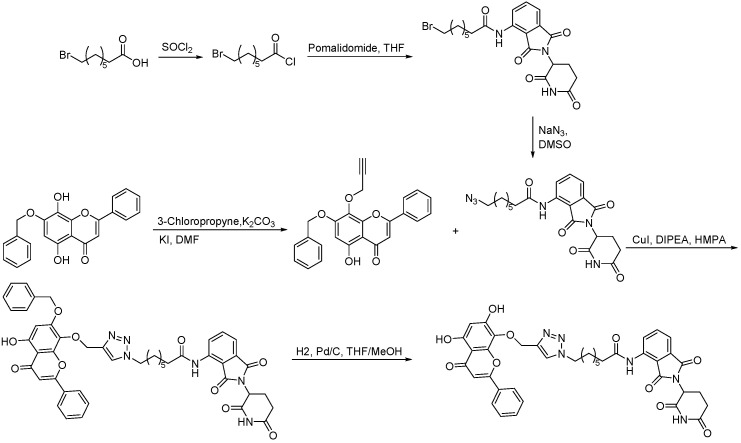
Synthesis of wogonin–based PROTAC.

**Scheme 6:**
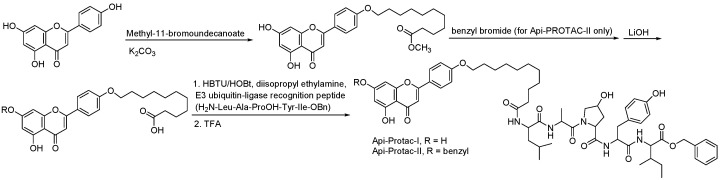
Synthesis of apigenin–based PROTAC.

**Scheme 7:**
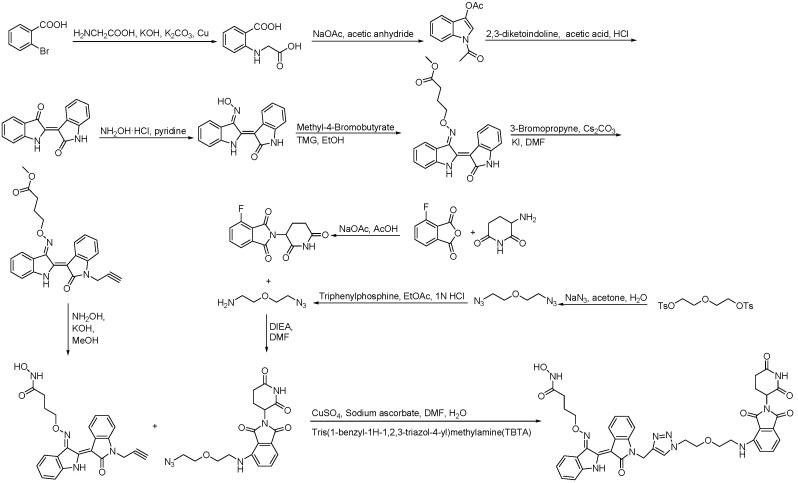
Synthesis of indirubin–based PROTAC.

**Scheme 8:**
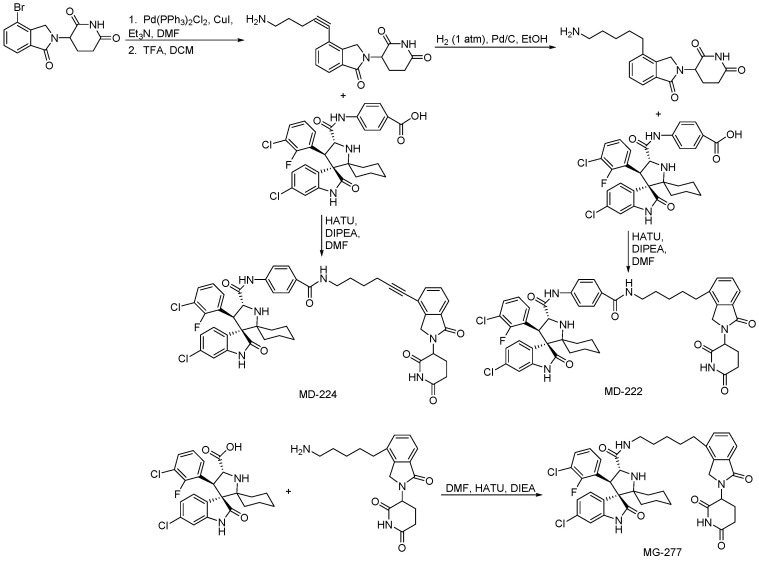
Synthesis of MI-1061– and MI-2103–based PROTAC.

**Scheme 9:**
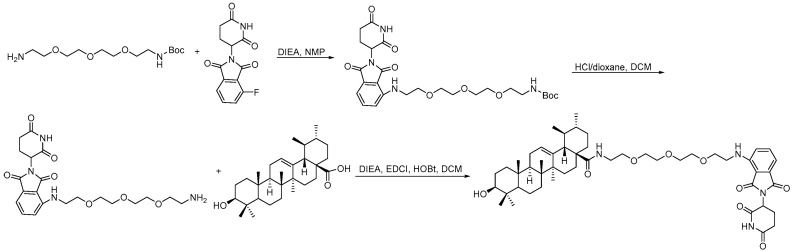
Synthesis of ursolic-acid–based PROTAC.

**Scheme 10:**
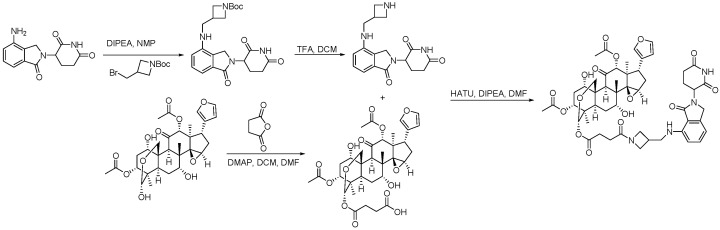
Synthesis of toosendanin–based PROTAC.

**Scheme 11:**
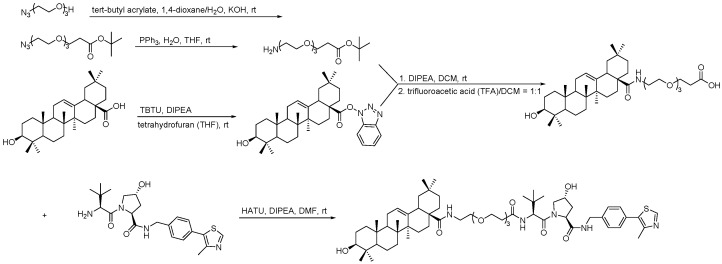
Synthesis of oleanolic-acid–based PROTAC.

**Scheme 12:**
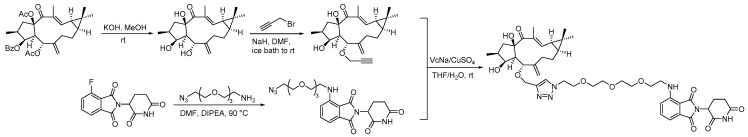
Synthesis of the lathyrol-derivatized–based PROTAC.

The efficacy of traditional Chinese medicine components and natural products includes realized through their interactions with multiple targets, and their affinities with target proteins are not very strong. The complexity of these mechanisms of action brings great challenges to the identification of their targets. In view of the fact that PROTAC molecules can effectively and specifically degrade target proteins without strong affinity, recent studies have demonstrated the potential of PROTAC technology in identifying the targets of traditional Chinese medicine components and natural products. In an article in 2020, PROTAC technology was combined with a quantitative proteomic analysis to identify the unknown target of the multikinase inhibitor sorafenib [41]. Recently, the research team at Shenyang Pharmaceutical University creatively introduced PROTAC technology into the field of traditional Chinese medicine research [42]. The combination of the PROTAC technique with quantitative proteomics and the molecular interaction detection technique represented by a microscale thermophoresis (MST) assay were proposed. It is proved that V-maf musculoaponeurotic fibrosarcoma oncogene homolog F (MAFF) is the target of a series of lathyrane diterpenoids obtained from the traditional Chinese medicine *Euphorbia lathyris*, and it is clarified that the anti-inflammatory effect of the lathyrane diterpenoid ZCY020 is based on the Nrf2/HO-1 signaling pathway. The core skeleton of the ZCY-001 compound lathyrol (Table 3) is linked to thalidomide (an E3 ligase CRBN ligand) by a PEG linker, and the resulting PROTAC molecule ZCY-PROTAC strongly degrades MAFF in a dose-dependent manner (Table 4). The specific synthesis processes of the PROTACs discussed in this section are shown in Scheme 5, Scheme 6, Scheme 7, Scheme 8, Scheme 9, Scheme 10, Scheme 11 and Scheme 12.

**Table 3:** Natural products 3–11.

No.	Natural Products	Structure	Structure Classification	Source of Compounds	Reference
3	Apigenin	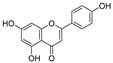	Flavonoid	Apple, celery, tea, fragrant plant, honey, etc	[26]
4	Wogonin	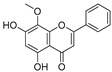	Flavonoid	*Scutellaria baicalensis*	[30]
5	MI-1061	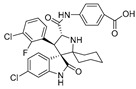	Alkaloid	Spirooxindole derivative	[35]
6	MI-2103	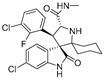	Alkaloid	Spirooxindole derivative	[36]
7	Ursolic acid	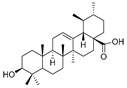	Triterpenoid	*Ligustrum lucidum Ait*	[37]
8	Indirubin	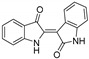	Bisindole alkaloid	Indigo naturalis	[38]
9	Toosendanin	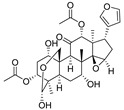	Triterpenoid	*Melia azedarach* L. and *Melia Toosendanin Sieb. et* Zucc	[39]
10	Oleanolic acid	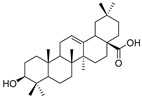	Triterpenoid	Olea europaea l	[40]
11	Lathyrol	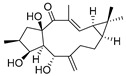	Lathyrane diterpenoids	*Euphorbia lathyris*	[42]

**Table 4:** Natural product targets 4–12.

No.	Target	E3	Cell Type	Reference
4	AhR	pVHL	Immortalized mouse hepatocyte cells and CV-1 cells (monkey kidney cell line)	[26]
5	CDK9	CRBN	MCF-7 and L02 cells	[30]
6	MDM2	CRBN	RS4;11 cells	[35]
7	MDM2	CRBN	RS4;11, MOLM-13, MDA-MB-468, MV-4-11, HL-60, and MDA-MB-231 cells	[36]
8	MDM2	CRBN	A549, Huh7, and HepG2 cells	[37]
9	HDAC6	CRBN	K562, HeLa, and THP-1 cells	[38]
10	STAT3	CRBN and VHL	CAL33 and HCT116 cells	[39]
11	Hemagglutinin protein	CRBN and VHL	Human embryonic kidney 293T cells	[40]
12	MAFF	CRBN	Mouse RAW264.7 macrophage and human embryonic kidney 293T (HEK293T) cells	[42]

### 2.3. Derived from Vitamins

**Scheme 13:**
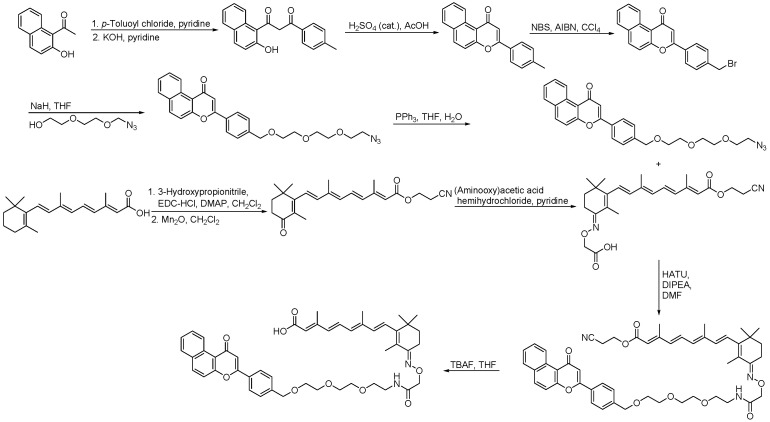
Synthesis of retinoic-acid–based PROTAC.

**Scheme 14:**
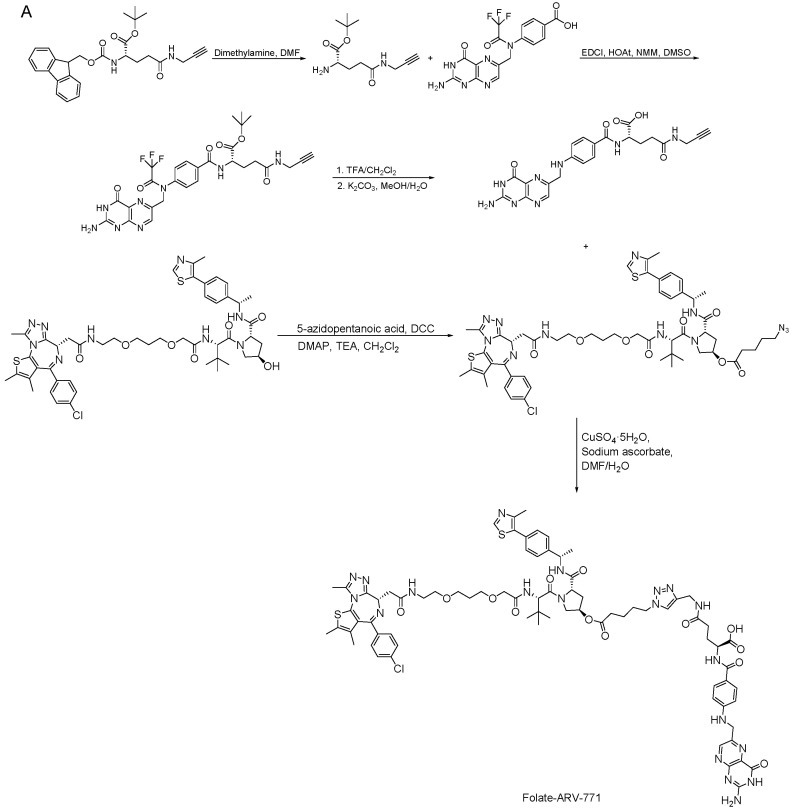

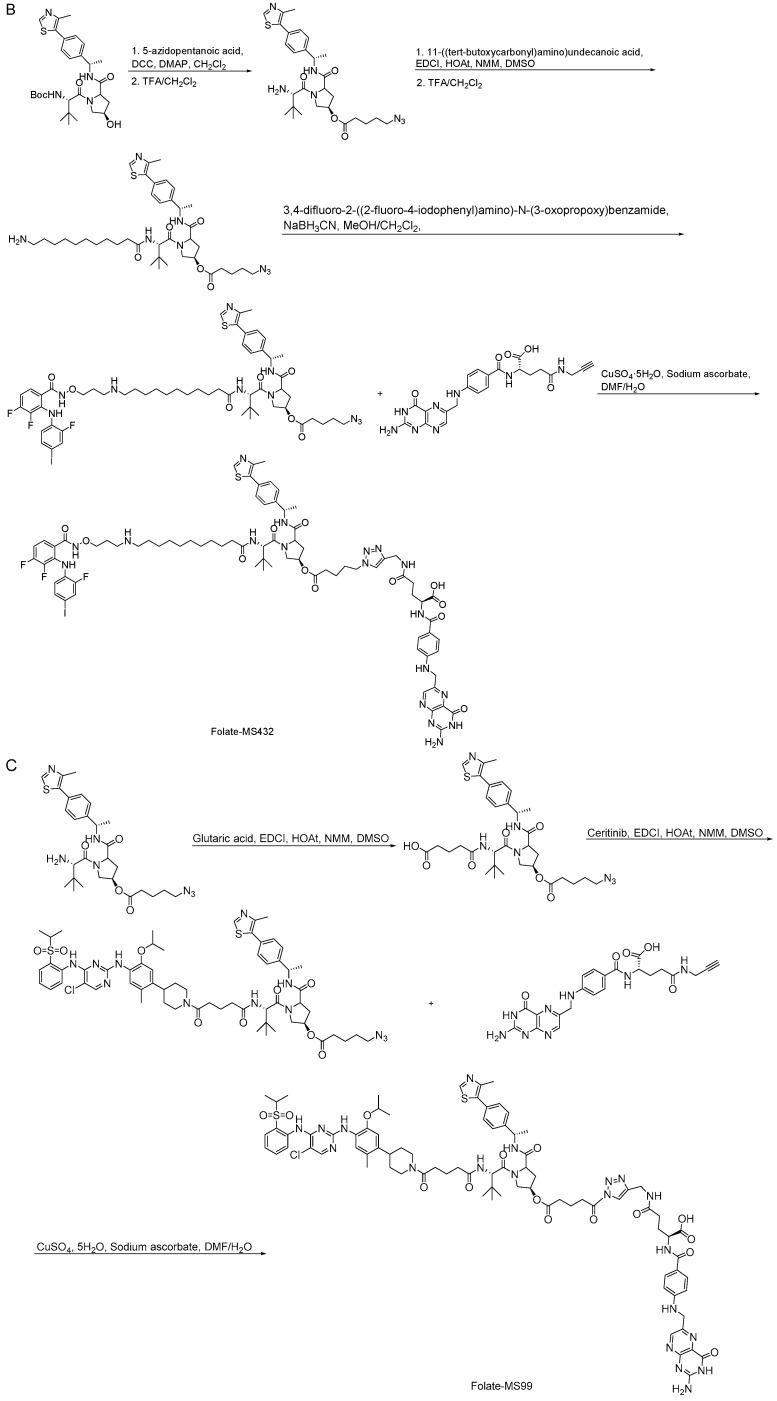
Synthesis of folate–based PROTACs. (**A**) Synthesis of folate-ARV-771. (**B**) Synthesis of folate-MS432. (**C**) Synthesis of folate-MS99.

Retinoic acid (RA) is a metabolic intermediate of vitamin A (retinol) in the body, and cellular retinoic-acid-binding proteins are important regulators of RA activity (Table 5) [43]. Studies have shown that an increased level of cellular retinoic-acid-binding protein 1 (CRABP1) in tumor cytoplasm is associated with RA resistance and that an increased level of cellular retinoic-acid-binding protein 2 (CRABP2) in the nucleus is associated with RA sensitivity, so RA is a potential tumor therapeutic target [44]. Studies have shown that the use of the Cullin4B-Ring (CRL4B) E3 ligase complex component AhR to recruit degradants and a small-molecule chimera composed of all-trans retinoic acid and AhR ligands connected by a suitable linker chain can effectively degrade CRABPs (Table 6) [45]. In addition, natural products can also be used as tools for the targeted delivery of PROTACs in vivo to achieve the selective degradation of target proteins in cancer cells. Folic acid, a water-soluble B vitamin, was first extracted and isolated from spinach leaves. Folic acid (Table 5) deficiency may induce cancer [46]. Folate receptor alpha (FOLR1) is highly expressed in a variety of cancers, including ovarian, lung, and breast cancers, but it has low or no expression in normal tissues and cells. FOLR1 is the most well-defined target for drug delivery to cancer cells, and other receptors, such as FOLR2 and FOLR3, have less affinity for folic acid than FOLR1 [47]. Based on PROTACs, a FOLR1-targeted drug delivery strategy is provided. A folic acid group is installed on the E3 ubiquitin ligase ligand. After entering cancer cells, folic acid-PROTAC is preferentially transported to cancer cells with high FOLR1 expressions. Intracellular hydrolase-catalyzed release and subsequently released PROTACs designed for specific target proteins recruit endogenous VHL E3 ubiquitin ligases to efficiently ubiquitinate and degrade cancer-cell-associated BRD, MEK, and ALK proteins (Table 6) [48]. The specific synthesis processes of the PROTACs discussed in this section are shown in Scheme 13 and Scheme 14.

### 2.4. Derived from Microorganisms

**Scheme 15:**
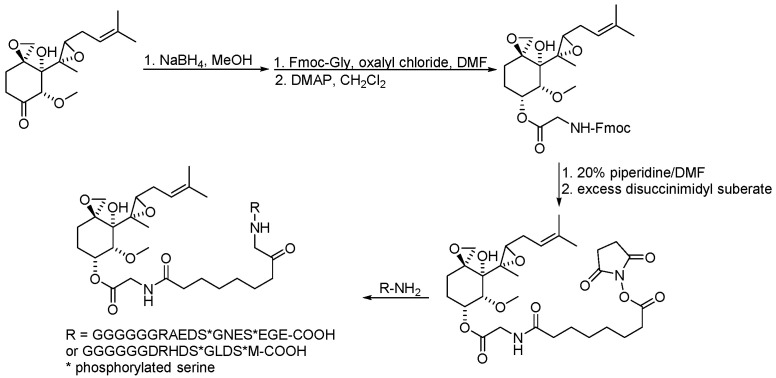
Synthesis of ovalicin–based PROTAC.

**Scheme 16:**
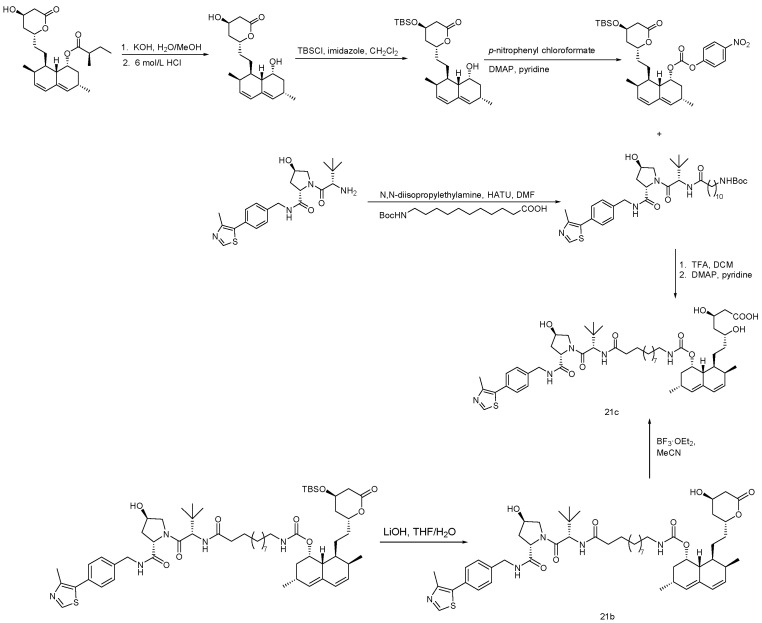
Synthesis of lovastatin–based PROTAC.

Ovalicin (Table 7) is one of the earliest microbial-derived immunosuppressants produced by fungi, and it has a strong inhibitory effect on lymphocyte proliferation and the DNA synthesis of lymphoma cells [49]. Ovalicin can covalently inhibit MetAP2, which plays a catalytic role in the cleavage of the N-terminal methionine of newly synthesized proteins in cells, thus regulating the growth cycle of endothelial cells and inhibiting tumor angiogenesis and growth [50]. Ovalicin was selected as the ligand of the MetAP2 protein, and the first PROTAC for the ubiquitination labeling and degradation of MetAP2 was designed and synthesized by properly binding to the phospopeptide binding to SKp1-Cullin-Fbox (SCF) of the ubiquitin ligase E3 system (Table 8) [51]. Because of the reduced efficacy of 3-hydroxy-3-methylglutaryl coenzyme A reductase (HMG-CoA reductase, HMGCR) accumulation after statin treatment, researchers found that, by inducing the degradation of HMGCR, the accumulation of HMGCR can be eliminated and cholesterol can be reduced [52]. Inspired by PROTACs, researchers designed and synthesized the PROTAC molecule P22A based on the E3 ligase CRBN. P22A can promote the expression of endogenous HMGCR proteins in Insig-1- and Insig-2-deficient Chinese hamster ovary (CHO) cells (SRD15 cells) [53]. The HMGCR inhibitor lovastatin (Table 7), which is also derived from microorganisms, is a drug used for the treatment of hypercholesterolemia. It can prevent the development of atherosclerosis, can reduce the incidence of myocardial infarction, and has a good effect on cardiovascular diseases [54]. Using lovastatin acid and VHL ligand conjugation, a powerful HMGCR-targeting PROTAC (21c) was identified that efficiently degrades HMGCR in Insig-silenced HepG2 cells (DC50 = 120 nmol/L). Furthermore, the corresponding lactone prodrug 21b has been shown to afford high plasma exposures referring to the active ingredient 21c, leading to efficient HMGCR degradation and promising cholesterol-lowering potency in vivo (Table 8) [55]. Microbial metabolites can be important sources for discovering natural PROTAC molecules. Recently, the first PROTAC molecule derived from a microbial natural product was reported [56]. The microbial natural product APL-16-5 (Table 7), based on the PROTAC mechanism, simultaneously binds to the E3 ubiquitin ligase TRIM25 and influenza virus PA subunit in order to induce TRIM25-dependent PA ubiquitination and degradation (Table 8). The TRIM25/APL-16-5/PA complex blocks viral RNA replication and has anti-influenza A virus activity in vivo. Apl-16-5 and its derivatives can also be used as TRIM25 ligands to further expand their applications. In addition, researchers suggest that the phenalenone fragment mainly contributes to interactions with PA and that the diterpene fragment determines the binding to TRIM25. The specific synthesis processes of the PROTACs discussed in this section are shown in Scheme 15 and Scheme 16.

### 2.5. Derived from Peptides

PROTACs designed using peptides to recruit target proteins have certain limitations in applications. However, PROTACs based on peptides are not only easy to modify but also have large protein-protein interaction surfaces, which have certain therapeutic potential for oncoproteins with unknown interactions [57,58]. β-catenin is an extremely attractive cancer target that plays an important role in the Wnt/β-catenin signaling pathway. As a β-catenin-targeted stapled peptide, xStAx (sequence: Ac-RRWPRSILDSHVRRVWR-NH_2_), derived from the tumor suppressor gene Axin, has been found for the first time to target and degrade β-catenin [59]. The xStAx-VHLL obtained by coupling xStAx with the VHL ligand not only promoted the continuous degradation of β-catenin in the tumor cells of various mouse tumor models but also strongly inhibited Wnt signaling. In addition, xStAx-VHLL also has a significant inhibitory effect on the survival of tumor organoids from patients with colorectal cancer. Researchers designed a cell-permeable peptide-based PROTAC. It was based on the homodimerized leucine-zipper-like motif in the C-terminus domain of the cell-cycle-related and expression-elevated proteins in tumors (CREPT, also named RPRD1B) to induce their degradation in vivo. In pancreatic cancer cells, PROTACs can induce CREPT degradation in a proteasome-dependent manner. Therefore, the use of protein-based dimeric structure interaction motifs may be a new approach to designing PROTACs [60].

**Scheme 17:**
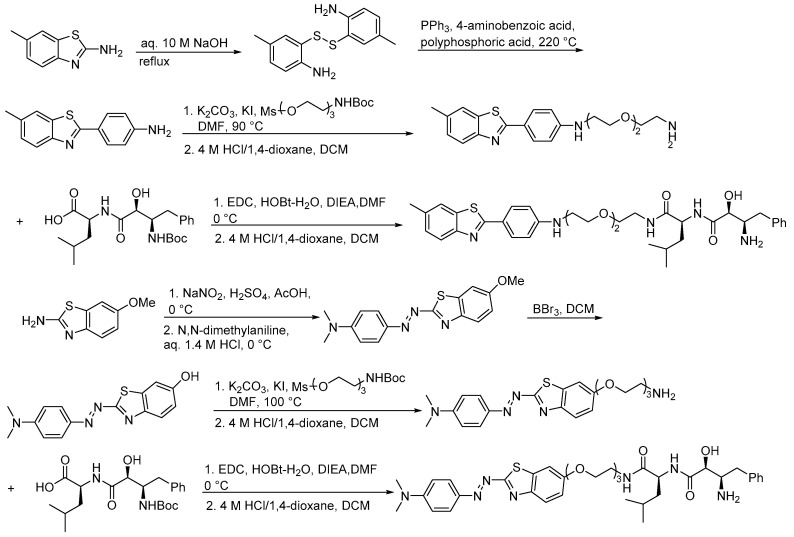
Synthesis of bestatin–based PROTAC.

**Scheme 18:**
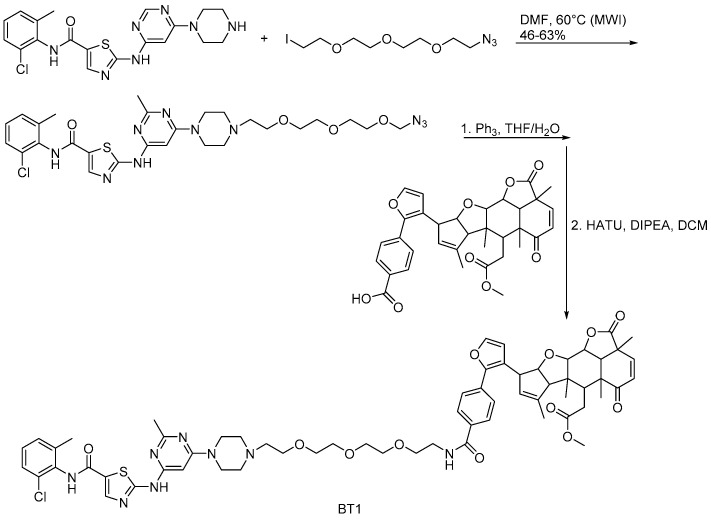
Synthesis of nimbolide–based PROTAC.

In addition, natural products and their derivatives can also be used as ligands to recruit E3 ubiquitin ligases in PROTACs. Bestatin is a natural product derived from streptomyces olivaceus (Table 9). Studies have shown that small hybrid molecules composed of amide derivatives of bestatin and polymeric probes induce Huntingtin protein (Htt) aggregates in Huntington’s disease (HD) to form complexes with the E3 ubiquitin ligase cIAP1, thereby leading to the proteasomal degradation of mHtt, and this approach can also be used to target other aggregation-prone proteins responsible for neurodegenerative diseases, including Alzheimer’s disease (AD), Parkinson’s disease (PD), and other polyQ (Table 10) [61]. Nimbolide, a triterpenoid derived from the leaves and flowers of *Azadirachta indica* L., can inhibit tumor genesis and metastasis (Table 9). Researchers found that the natural product nimbolide is a covalent ligand of the E3 ligase RNF114 via an activity-based proteome profiling (ABPP) platform, and they further confirmed that the recruitment of RNF114 by nimbolide can be used to target protein degradation (Table 10). The PROTACs formed by nimbolide coupled with BCR-ABL fused with the oncogene inhibitor dasatinib BT1 selectively degrade BCR-ABL but not c-ABL in leukemia cells, compared with previously reported CRBN- and VHL-recruited BCR-ABL inhibitors [62,63]. The specific synthesis processes of the PROTACs discussed in this section are shown in Scheme 17 and Scheme 18.

## 3. Molecular Glue Degradation Agents Derived from Natural Products

Protein-protein interactions induced by molecular glues also have great application potential in the field of protein degradation. Currently, molecular glues are being examined in clinical studies, but most of them were accidentally discovered, and there is no good drug development strategy at present, so the discovery of new molecular glues is very challenging. Ubiquitin ligases regulate a variety of plant hormone signaling pathways. Studies have shown that, when an F-box protein transport inhibitor response 1 (TIR1) protein binds to auxin (Table 11), it will initiate SCF ubiquitin ligase to degrade downstream auxin/indole-3-acetic acid (Aux/IAA) transcription factors. These proteins are assembled into SCFTIR1 protein complexes in plants. Studies have shown that auxin can regulate the degradation of Aux/IAA proteins by directly binding to TIR1 (Table 12). The regulatory mechanism of TIR1 by auxin suggests that there may be a small molecule in ubiquitin ligases that facilitates protein-protein interactions [64]. Researchers found that the leucine-rich repeat domain of TIR1 contains a phytate cofactor present in plants and eukaryotes (InsP6/phytate), which recognizes auxin and Aux/IAA polypeptide substrates through a single surface pocket [65].

Nomura’s team found that a manumycin polyketide, asukamycin (Table 11), has multiple electrophilic sites, targets Cys374 of the E3 ligase UBR7 in breast cancer cells, and participates in the molecular glue interaction of the new substrate tumor suppressor TP53 in order to form a UBR7-asukamycin-TP53 complex to exert anticancer effects (Table 12). Combined with chemical proteomic studies, it was found that asukamycin can also serve as a probe for the rational identification of molecular glues from electrophilic natural products [66]. Recently, a natural-product-derived molecular glue has been identified using a human proteome chip (Table 11). The study identified a natural-derived small-molecule bufalin that promotes the rapid degradation of E2F transcription factor 2 (E2F2) and inhibits hepatocellular carcinoma (Table 12). In addition, the pyrone structure of bufalin has been suggested as a potential structural group for targeting the atypical E3 ligase Zinc finger protein 91 (ZFP91). Its carbonyl-conjugated structure may possess electrophilic addition capability in order to covalently bind to the cysteine in ZFP91 [67].

## 4. Perspectives and Conclusions

**Figure 2:**
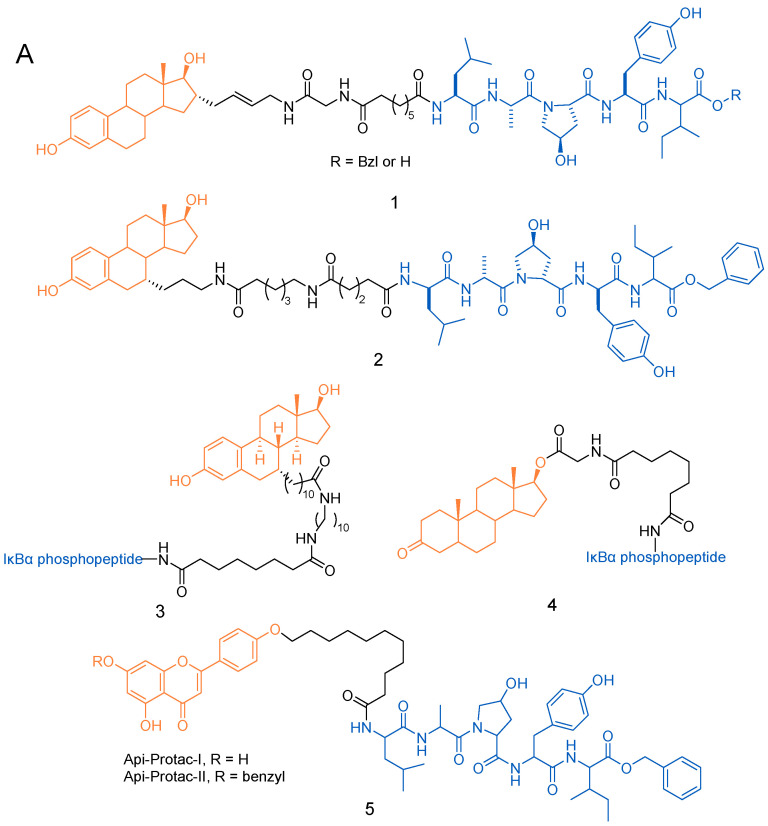

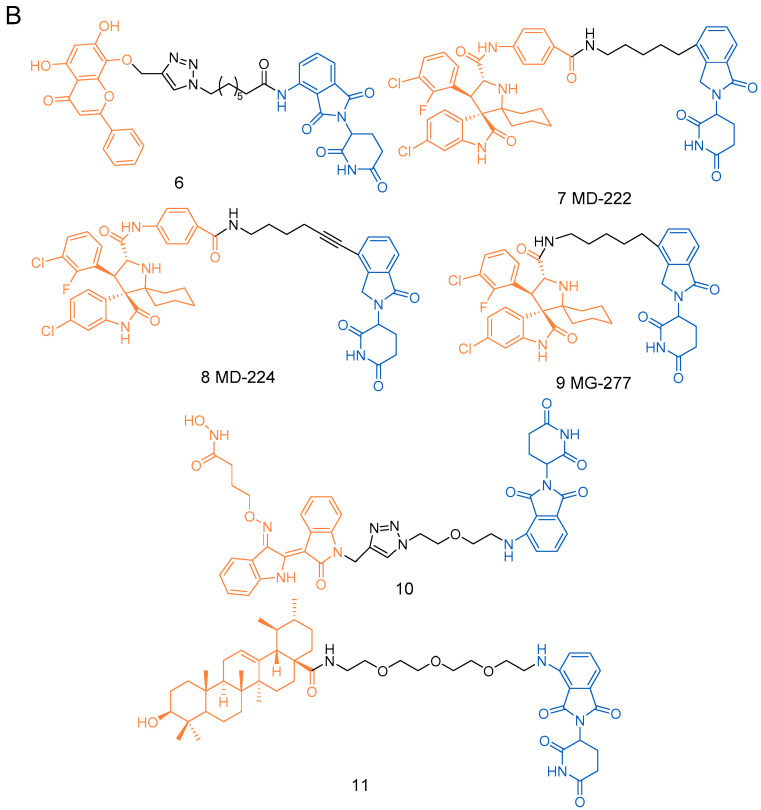

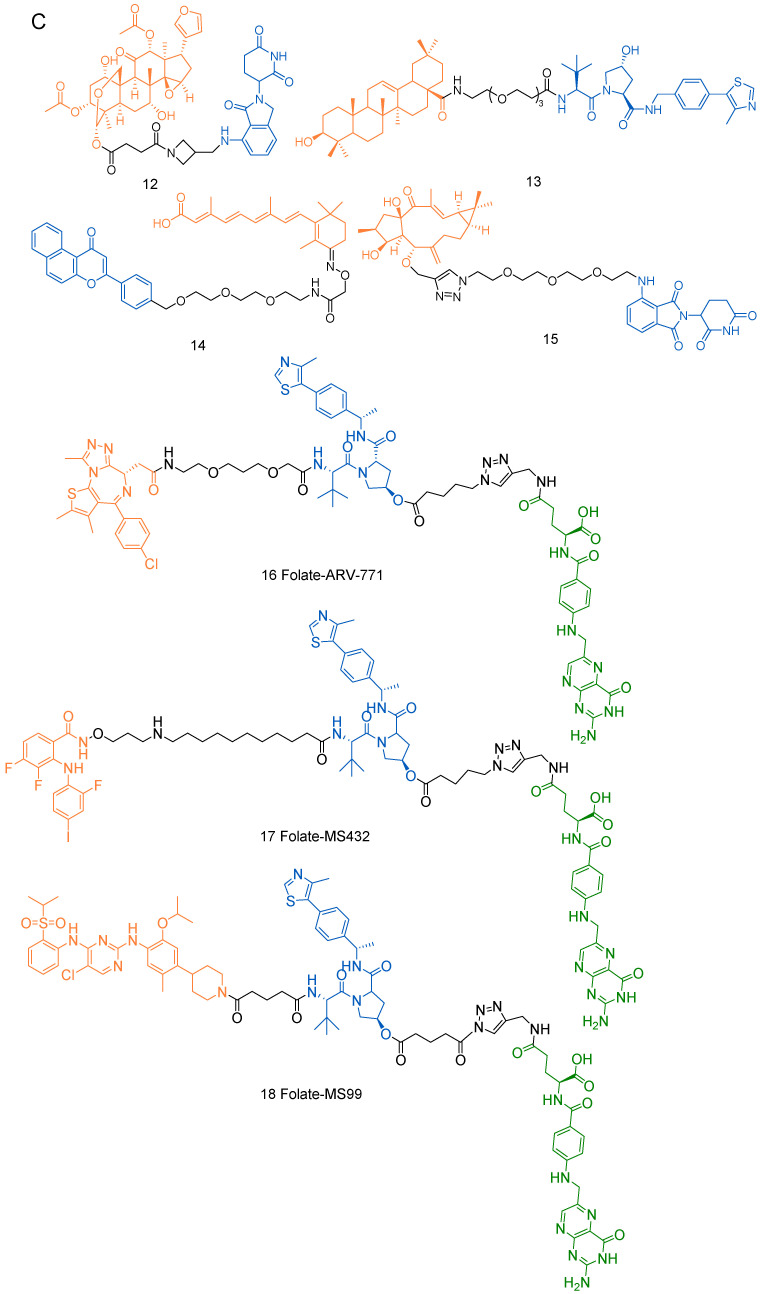

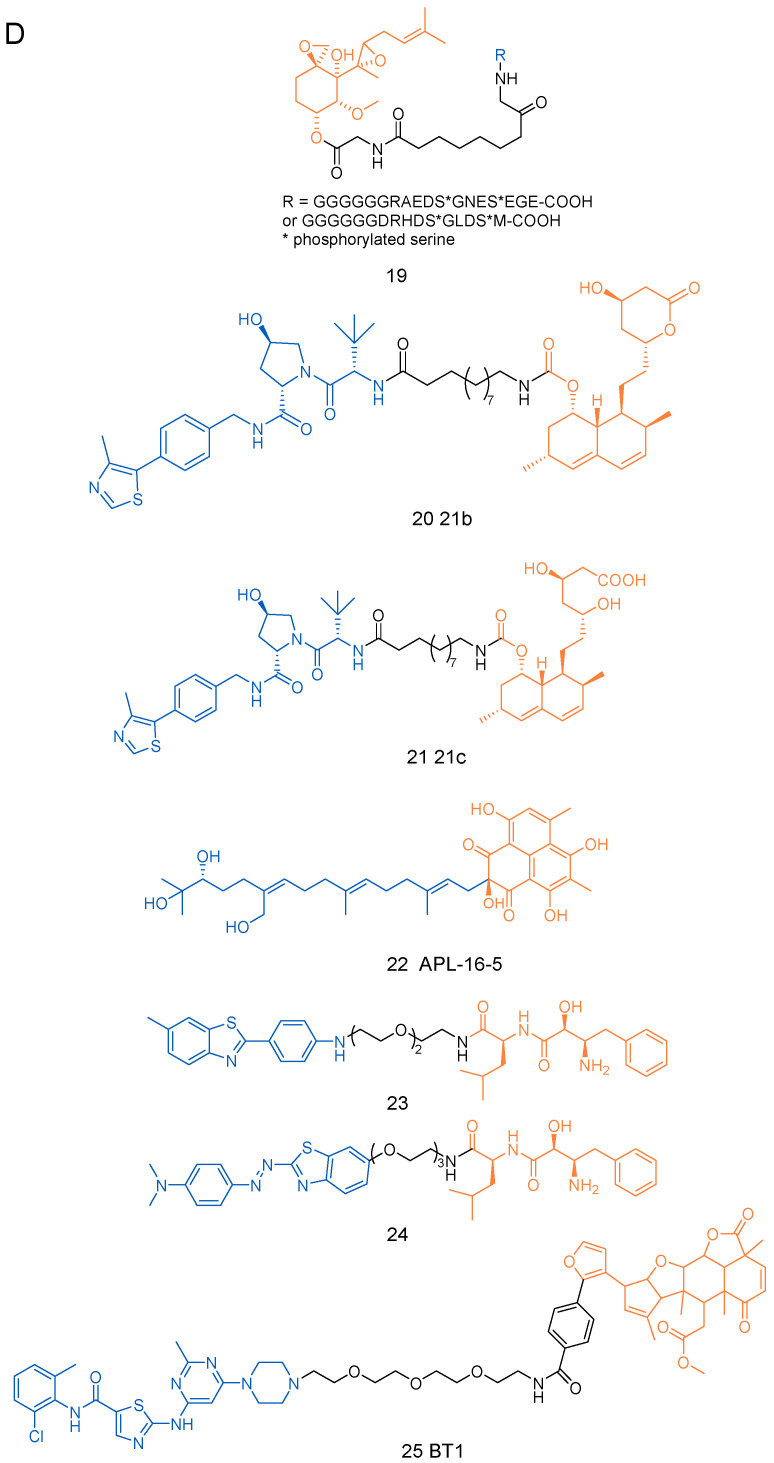
The structures of PROTACs presented in this review. (**A**) The structures of PROTACs 1–5f. (**B**) The structures of PROTACs 6–11f. (**C**) The structures of PROTACs 12-18f. (**D**) The structures of PROTACs 19–25f.

This review summarized the progress of protein-targeting degradation agents based on natural products, such as PROTACs and molecular glues (Figure 2). The classes of these natural products include hormones, flavonoids, alkaloids, terpenoids, vitamins, microorganisms, and peptides. In conclusion, PROTACs and molecular glues based on natural products and their derivatives have good application prospects in the targeted degradation of disease-related proteins. In addition, PROTACs mainly degrade intracellular proteins, but 40% of gene products are extracellular and membrane-associated proteins, such as growth factors, cytokines, and chemokines, which cause abnormalities in various diseases, such as pain and inflammation signaling. Lysosome-targeted chimera (LYTAC) is a degradation technology targeting extracellular proteins, and it was reported in *Nature* by the research team of Professor Bertozzi of Stanford University. The study confirmed that LYTAC successfully degraded the epidermal growth factor receptor (EGFR), programmed death-ligand 1 (PD-L1), and apolipoprotein E4 [68]. Stanford researchers have also shown that LYTACs can target and degrade important proteins in Alzheimer’s disease and cancer in cells. The structure of an LYTAC is similar to that of a PROTAC. One side of an LYTAC is an oligopeptide group that can bind to the transmembrane receptor CI-M6PR on the cell surface, the other side is an antibody or small molecule that can bind to the target protein, and the two sides are connected by a linker. For example, LYTAC AB-2 is a conjugation of the anti-EGFR monogram lowering antibody cetuximab and the CI-M6PR ligand M6Pn sugar polypeptide. The complex can be engulfed by the cell membrane to form transport vesicles and then transferred to the lysosome for degradation under the action of CI-M6PR. The receptor CI-M6PR can be recycled and returned to the cell membrane in order to degrade EGFR. In addition, there are other emerging targeting protein technologies, such as light-controlled target protein degradation (photo-PROTAC), autophagy-mediated target protein degradation (AUTAC), and autophagosome-tethering compounds (ATTECs). These techniques not only expand the pathway of protein degradation but also improve the accuracy of protein degradation.

In addition, signal transduction associated with disease is an extremely complex and multifactorial regulatory process. Therefore, single-target therapy is often insufficient to curb disease progression, and new multi-target drugs or drug combinations need to be designed to block related signal transduction for different pathways and mechanisms to achieve the effect of treating the disease and reduce the drug resistance [69,70]. Recently, novel dual PROTACs with gefitinib/olaparib and CRBN/VHL E3 ligands as substrates have been reported to degrade EGFR and PARP in cancer cells simultaneously [71]. As the first successful case of dual PROTACs, this technology will greatly broaden the application of PROTAC methods and open up a new field for drug discovery. In addition, recent studies have shown that natural products have structural diversity, high activity, and low toxicity and that their therapeutic effects are generally achieved through multi-target therapy [72]. Therefore, the use of natural products is one of the important ways to obtain multi-targeted drugs, and they are also a hot direction of multi-targeted drug research. Meanwhile, there has been increasing interest in the use of PROTAC and molecular glue strategies to recognize natural product target proteins. With the help of proteomics and other technologies, protein-targeted degradation agents based on natural products will have great application potential in the field of protein-targeted degradation.

## Figures and Tables

**Table 5:** Natural products 12–13.

No.	Natural Products	Structure	Structure Classification	Source of Compounds	Reference
12	Retinoic acid	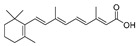	Retinol	Vitamin	[45]
13	Folate	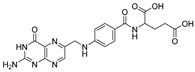	Pteroylglutamic acid	Vitamin	[48]

**Table 6:** Natural product targets 13–16.

No.	Target	E3	Cell Type	Reference
13	CRABPs	AhR E3 ligase complex	MCF-7 and IMR-32 cells	[45]
14	BRD	VHL	HeLa cells	[48]
15	MEK	VHL	HT-29 cells	[48]
16	ALK	VHL	SU-DHL-1 cells and HT-29 cells	[48]

**Table 7:** Natural products 14–16.

No.	Natural Products	Structure	Structure Classification	Source of Compounds	Reference
14	Ovalicin	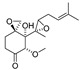	Sequiterpene	Pseudeurotium ovalis	[51]
15	Lovastatin	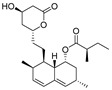	Poly ketone	Monascus	[55]
16	APL-16-5	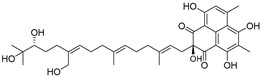	Asperphenalenone	Plant endophytic fungus Aspergillus sp. CPCC 400735	[56]

**Table 8:** Natural product targets 17–19.

No.	Target	E3	Cell Type	Reference
17	MetAP2	SCF^β-TRCP^	293T cells	[51]
18	HMGCR	VHL	HepG2 cells	[55]
19	Viral endonuclease PA	TRIM25	HEK293T, A549, MDCK, Huh7.5.1, Vero, and BHK21cells	[56]

**Table 9:** Natural products 17–18.

No.	Natural Products	Structure	Structure Classification	Source of Compounds	Reference
17	Bestatin	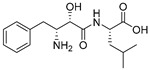	Dipeptide	Streptomyces olivoreticuli	[61]
18	Nimbolide	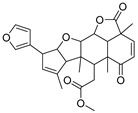	Triterpenoid	Veda neem leaf	[62]

**Table 10:** Natural product targets 20–21.

No.	Target	E3	Cell Type	Reference
20	mHtt	cIAP1	Hela cells	[61]
21	BCR-ABL	RNF114	K562 cells	[62]

**Table 11:** Natural products 19–21.

No.	Natural Products	Structure	Structure Classification	Source of Compounds	Reference
19	Auxin	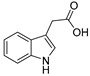	Indole Acetic Acid	Phytohormone	[64]
20	Asukamycin	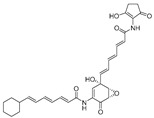	Polyketide	*Streptomyces nodosus*	[66]
21	Bufalin	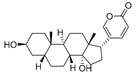	Steroids	Venenum Bufonis	[67]

**Table 12:** Natural product targets 22–24.

No.	Target	E3	Cell Type	Reference
22	Aux/IAA	TIR1	Hi5 suspension insect cells	[64]
23	TP53	UBR7	231MFP cells	[66]
24	E2F2	ZFP91	HepG2 cells	[67]

## Data Availability

Not applicable.
